# Karyotypic diversity in seven Amazonian anurans in the genus *Hypsiboas* (family Hylidae)

**DOI:** 10.1186/1471-2156-15-43

**Published:** 2014-04-04

**Authors:** Thais Lemos de Mattos, Ana Carolina Coelho, Carlos Henrique Schneider, David Otávio Carmo Telles, Marcelo Menin, Maria Claudia Gross

**Affiliations:** 1 Instituto Nacional de Pesquisas da Amazônia, Programa de Pós-Graduação em Genética, Conservação e Biologia Evolutiva, Av. André Araújo, 2936, 69080-971 Manaus, AM, Brazil; 2 Universidade Federal do Amazonas, Instituto de Ciências Biológicas, Departamento de Biologia, Laboratório de Citogenômica, Av. General Rodrigo Octávio Jordão Ramos, 3000, 69077-000 Manaus, AM, Brazil; 3 Universidade Federal do Amazonas, Instituto de Ciências Biológicas, Programa de Pós-Graduação em Diversidade Biológica, Av. General Rodrigo Octávio Jordão Ramos, 3000, 69077-000 Manaus, AM, Brazil

**Keywords:** *Hypsiboas* groups, Chromosomes, Heterochromatin, Nucleolar organizer region, Telomere

## Abstract

**Background:**

*Hypsiboas* species have been divided into seven groups using morphological and genetic characters, but for most of the species, there is no cytogenetic information available. A cytogenetic analysis using conventional staining, C-banding, silver staining, and fluorescence in situ hybridization (FISH) with telomeric sequence probes were used to investigate the karyotype of seven Amazon species of the genus *Hypsiboas* belonging to the following intrageneric groups: *H. punctatus* (*H. cinerascens*), *H. semilineatus* (*H. boans*, *H. geographicus*, and *H. wavrini*), and *H. albopunctatus* (*H. lanciformis*, *H. multifasciatus*, and *H. raniceps*). The aim was to differentiate between the karyotypes and use the chromosomal markers to distinguish between the *Hypsiboas* groups. The data were compared with a previous phylogenetic proposal for these anurans. In addition, *H. lanciformis*, *H. boans*, and *H. wavrini* are described here for the first time, and we characterize the diploid numbers for *H. cinerascens*, *H. geographicus*, *H. multifasciatus*, and *H. raniceps.*

**Results:**

The diploid number for all of the species analyzed was 24, with the exception of *Hypsiboas lanciformis*, which had 2n = 22 chromosomes. The constitutive heterochromatin distribution, nucleolar organizer region locations, and interstitial telomeric sites differed between the species. A hypothesis that the heterochromatic patterns are evolving is proposed, with the divergence of the groups probably involving events such as an increase in the heterochromatin in the species of the *H. semilineatus* group. The FISH conducted with the telomeric probes detected sites in the terminal regions of all of the chromosomes of all species. Interstitial telomeric sites were detected in three species belonging to the *H. semilineatus* group: *H. boans*, *H. geographicus*, and *H. wavrini.*

**Conclusion:**

The results of this study reinforce the complexity previously observed within the genus *Hypsiboas* and in the different groups that compose this taxon. More studies are needed focusing on this group and covering larger sampling areas, especially in the Brazilian Amazon, to improve our understanding of this fascinating and complex group.

## Background

Hylidae is considered the most diverse family among the anurans, with 936 described species [1], of which about 90 are found in the Brazilian Amazon [2]. Recent cytogenetic studies of species from this family have demonstrated intrapopulational variation, with polymorphisms of the nucleolar organizer regions (NORs) [3], different diploid numbers in the same nominal species [4,5] and intra-generic variations such as the localization of the NORs among species [6].

Based on a compilation of cytogenetic data for the hylids, the majority of the species had a diploid number of 26 [7], although some genus such as the *Hypsiboas* spp. showed reductions, with the majority having 2n = 24 chromosomes [8-12]. Despite the conserved constant diploid number found in *Hypsiboas* spp., the karyotypic organization of the species cannot be considered conserved (Table 1) [4-30].

**Table 1:** **Review of cytogenetic data available in the literature for *Hypsiboas *** **species**

**GR**	**Specie**	**Locality**	**2n**	**CF**	**NF**	**C-banding**	**NOR (pair)**	**Reference**
*H. albopunctatus***group**	*Hypsiboas albopunctatus*	Rio Claro (SP)	22	6m + 6sm + 10st	44	Centromeric	8	[4]
		Rio Claro (SP)	22 + 1B	6m + 6sm +10st + 1B	45	-	8	[4]
		-	22	-	-	-	-	[9]
		Pirenópolis (GO)	22	10m + 4sm + 8sm	44	-	-	[12]
		-	22	-	-	-	-	[14]
	*Hypsiboas lanciformis*	Manaus (AM)	22	8m + 6sm + 8st	44	Centromeric in most of chromosomes	1,11	Present study
						Pericentromeric (pairs 1,3), short arm (pairs 4,11) and absent (pair 7)		
	*Hypsiboas multifasciatus*	Serranópolis (GO)	24	14m + 8sm + 2st	48	-	-	[12]
		Iranduba (AM)	24	10m + 6sm + 8st	48	Interstitial in most of chromosomes and in the long arms (pairs 11,12)	11	Present study
	*Hypsiboas raniceps*	Brasilândia (MT)	24	8m + 10sm + 6st	48	Almost absent	11	[4]
		-	24	-	-	-	-	[10]
		Tangará da Serra (MT)	24	12m + 8sm + 4st	48	-	1,11	[11]
		-	24	-	-	-	-	[15]
		Iranduba (AM)	24	10m + 6sm + 8st	48	Absent and pericentromeric (pair 5)	11	Present study
*H. faber***group**	*Hypsiboas albomarginatus*	Bertioga; (SP)	24	18m + 6sm	48	Centromeric	2	[6]
		Picinguaba (SP)	24	18m + 6sm	48	Centromeric	2	[6]
		-	24	-	-	-	-	[9]
		-	24	-	-	-	-	[16]
		Cariacica (ES)	24	12m + 10sm + 2st	48	-	2	[17]
		Anchieta (ES)	24	12m + 10sm + 2st	48	-	2	[17]
	*Hypsiboas crepitans*	Piranhas (AL)	24	8m + 4sm + 12st	48	Centromeric	11	[4]
		-	24	-	-	-	-	[8]
		-	24	-	-	-	-	[10]
		-	24	-	-	-	-	[14]
		-	24	8m + 4sm + 10st	-	Telomeric	-	[18]
	*Hypsiboas faber*	Mogi das Cruzes (SP)	24	12m + 8sm	48	-	11	[6]
		Biritiba-Mirim (SP)	24	12m + 8sm	48	-	11	[6]
		-	24	-	-	-	-	[9]
		Pedra Azul (ES)	24	8m + 8sm + 8st	48	-	11	[17]
	*Hypsiboas lundii*	Pirenópolis (GO)	24	14m + 6sm + 4st	48	-	--	[12]
	*Hypsiboas pardalis*	-	24	-	-	-	--	[14]
		Cariacica (ES)	24	10m + 10sm + 4st	48	-	11	[17]
	*Hypsiboas rosenbergi*	-	24	-	-	-	-	[19]
*H. pellucens***group**	*Hypsiboas rufitelus*	-	24	-	-	-	-	[20]
*H. pulchellus***group**	*Hypsiboas bischoffi*	Rancho Queimado (SC)	24	-	-	-	10	[9]
		-	24	-	-	-	-	[21]
		-	24	-	-	-	-	[22]
	*Hypsiboas caingua*	-	24	-	-	-	-	[23]
	*Hypsiboas cordobae*	Cordoba (ARG)	24	6m + 6sm	48	Centromeric		[24]
		San Luis (ARG)	24	6m + 6sm	48	Centromeric		[24]
	*Hypsiboas guentheri*	Rancho Queimado (SC)	24	-	-	-	10	[22]
	*Hypsiboas joaquini*	-	24	-	-	-	-	[23]
	*Hypsiboas marginatus*	São Francisco de Paula (RS)	24	10m + 10sm + 4st	48	Centromeric	10	[25]
	*Hypsiboas polytaenius*	-	24	-	-	-	-	[10]
		-	24	-	-	-	-	[15]
		-	24	-	-	-	-	[14]
		Santa Teresa (ES)	24	12m + 10sm + 2st	48			[26]
	*Hypsiboas prasinus*	-	24	-	-	-	-	[9]
		-	24	-	-	-	-	[23]
		Serra do Japi (SP)	24	8m + 10sm + 6st	48	Centromeric	9,12	[27]
	*Hypsiboas pulchellus*	-	24	-	-	-	-	[9]
		-	24	-	-	-	-	[14]
		-	24	-	-	-	-	[23]
		Cordoba (ARG)	24	6m + 6sm	48	Pericentromeric		[24]
		-	24	-	-	-	-	[28]
	*Hypsiboas semiguttatus*	-	24	-	-	-	-	[21]
		Camabará do Sul (SC)	24	10m + 10sm + 4st	48	Centromeric	1	[25]
		São Francisco de Paula (RS)	24	10m + 10sm + 4st	48	Centromeric	1	[25]
		Piraquara (PR)	24	10m + 10sm + 4st	48	Centromeric	1	[25]
*H. punctatus***group**	*Hypsiboas cinerascens*	-	24	-	-	-	-	[14]
		Manaus (AM)	24	6m + 12sm + 6st	48	Centromeric (pairs 1,2,3,5,6,8) and poorly distinguishable in most of the chromosomes	11	Present study
	*Hypsiboas punctatus*	-	24	-	-	-	-	[14]
		-	24	-	-	-	-	[21]
		-	24	-	-	-	-	[29]
		-	24	-	-	-	-	[30]
*H. semilineatus***group**	*Hypsiboas boans*	São Sebastião do Uatumã (AM)	24	8m + 6sm + 10st	48	Centromeric and pericentromeric regions	11	Present study
	*Hypsiboas geographicus*	-	24	-	-	-	-	[21]
		Santa Isabel do Rio Negro (AM)	24	10sm + 6sm + 8st	48	Centromeric and pericentromeric regions, no distinguishable (pairs 6,7)	-	Present study
	*Hypsiboas* gr. *geographicus*	-	24	-	-	-	-	[29]
	*Hypsiboas semilineatus*	Santa Teresa (ES)	24	10m + 6sm + 8st	48	-	11	[17]
	*Hypsiboas wavrini*	Santa Isabel do Rio Negro (AM)	24	10m + 6sm + 8st	48	Centromeric and pericentromeric regions	11	Present study
		São Sebastião do Uatumã (AM)	24	10m + 6sm + 8st	48	Centromeric and pericentromeric regions	11	Present study

Species are allocated according to the group (GR) to which they belong. The collection site (Locality), diploid number (2n), chromosome formula (CF), fundamental number (FN), constitutive heterochromatin distribution pattern (C-banding), and nucleolus organizer regions (NORs) are indicated. A dash (-) signifies the data was not included in the publication.

The species of the *Hypsiboas* genus have been separated into seven large species groups: *H. albopunctatus*; *H. benitezi*; *H. faber*; *H. pellucens*; *H. pulchellus*; *H. punctatus*; and *H. semilineatus*[13,17,31]. This classification was suggested to reflect a number of distinct morphological characters among the species, principally coloration, size, the presence of interdigital membranes or spines on the prepollex of the males [32-34], and synapomorphies among their molecular markers [13]. According to a phylogeny proposal [13] for the consensus tree, all groups are considered monophyletic, and the *H. punctatus* group is a sister group separate from the other groups. *H. pulchellus* and *H. faber* are sister groups, as are *H. pellucens* and *H. albopunctatus.* The group [*H. pulchellus* + *H. faber*] is a sister to [*H. pellucens* + *H. albopunctatus*], and these four species groups are a sister of *H. semilineatus. Hypsiboas cinerascens* (previously *Hyla granosa* in the *Hyla granosa* group) belongs to the *Hypsiboas punctatus* group (a monophyletic group fusion between the *Hyla punctata* and *Hyla granosa* groups) and *H. wavrini* is not included in the phylogeny because not all species are used to build a phylogenetic tree [13].

Regarding the karyotypic descriptions available for the species composing the *H. albopunctatus* group, there is some degree of confusion about the names adopted for the different taxa, resulting in divergences of the cytogenetic information available. Only *H. albopunctatus*, *H. fasciatus*, and *H. raniceps* have been karyotyped [4,9-11,15,31] even though reportedly there was no cytogenetic data available for *H. fasciatus*[31]. *H. multifasciatus* was cytogenetically described for the first time by Beçak [31]. However, Beçak [9] did not describe *H. multifasciatus*, but rather *H. bischoffi*, which belongs to the *H. pulchellus* group and is similar to *H. multilineatus*[34]. This indicates that the *H. albopunctatus* group may comprise species complexes [34], which would explain why different cytotypes have been described for the same species—such as *H. multifasciatus* from the states of Amazonas and Goiás in Brazil [12]. A similar situation was observed in *H. raniceps*, which has been described as having three distinct karyotypic formulas among the individuals encountered in the states of Mato Grosso and Goiás in central western Brazil [4,11], with one additional formula from Amazonas in northern Brazil.

There are both inter- and intraspecific variations in the chromosome formulas in the positions of their nucleolus organizer regions (NORs) and in the distribution of the constitutive heterochromatin [4,8,9,16-18,24,25,27]. Additionally, the species *H. albopunctatus* demonstrates a reduction in the diploid number, having 2n = 22 chromosomes in addition to the presence of a B chromosome [4,12]. The karyotypic patterns of organization are not established for the groups, and it is impossible to know if there are any cytogenetic features that characterize the *Hypsiboas* groups or if a concordance between the phylogenetic proposal and the chromosomal patterns exists. Thus, the objective of this study was to cytogenetically characterize one species of the *H. punctatus* group (*H. cinerascens*); three species of the *H. semilineatus* group (*H. boans*, *H. geographicus*, and *H. wavrini*), and three species of the *H. albopunctatus* group (*H. lanciformis*, *H. multifasciatus*, and *H. raniceps*) that occur in Amazonas, Brazil and to distinguish the *Hypsiboas* groups using chromosomal markers. In addition, we compared the results with Faivovich et al.’s phylogenetic proposal [13]. This manuscript is the first to describe *H. lanciformis*, *H. boans*, and *H. wavrini*, and we additionally characterize the diploid numbers for *H. cinerascens*, *H. geographicus*, *H. multifasciatus*, and *H. raniceps*.

## Results

### Diploid number, fundamental number and chromosomal formula

**Figure 1:**
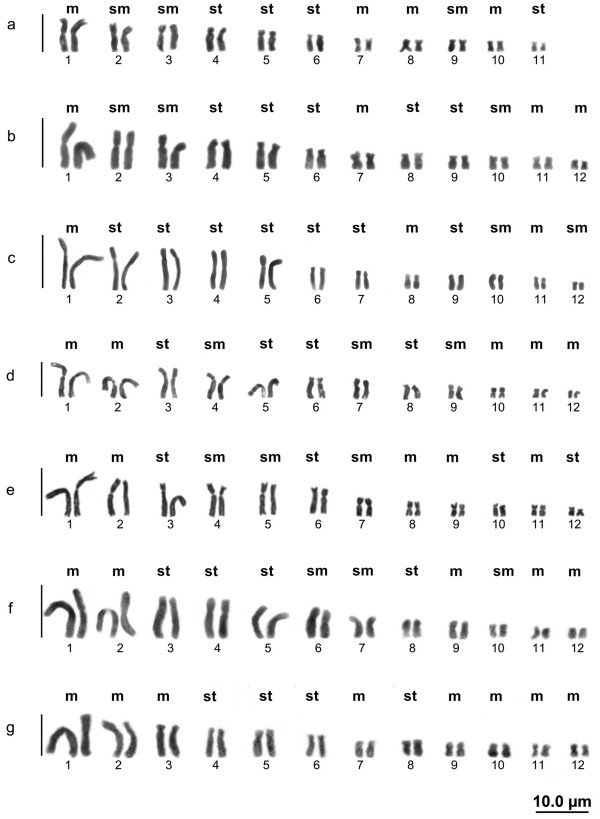
**Mitotic karyotypes using conventional staining with Giemsa. ***Hypsiboas lanciformis ***(a)**; *H. boans ***(b)**; *H. cinerascens ***(c)**; *H. geographicus ***(d)**; *H. multifasciatus ***(e)**; *H. raniceps ***(f)**; and *H. wavrini ***(g)**.

*Hypsiboas lanciformis* showed a diploid number of 22 (Figure 1a), while the species *H. boans* (Figure 1b), *H. cinerascens* (Figure 1c), *H. geographicus* (Figure 1d), *H. multifasciatus* (Figure 1e), *H. raniceps* (Figure 1f), and *H. wavrini* (Figure 1g) had 2n = 24 chromosomes, without any indication of sexual and/or supernumerary chromosomes. All of the species had a fundamental number (FN) of 48, with the exception of *H. lanciformis* (FN = 44).

The chromosomal formulas were different for the four species: *H. lanciformis*, 8m + 6sm + 8st; *H. boans*, 8m + 6sm + 10st; *H. cinerascens*, 6m + 12sm + 6st; and *H. wavrini*, 10m + 6sm + 8st. Three species, *H. geographicus*, *H. multifasciatus*, and *H. raniceps* had a chromosomal formula of 10m + 6sm + 8st.

### C-banding and staining of the silver‒binding nucleolar organizer region

**Figure 2:**
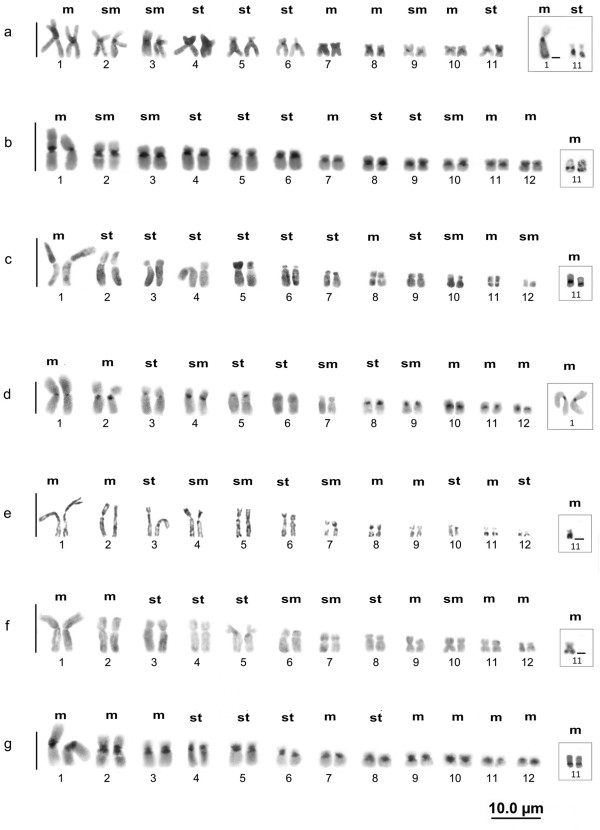
**Distribution patterns of the constitutive heterochromatin. ***Hypsiboas lanciformis ***(a)**; *H. boans ***(b)**; *H. cinerascens ***(c)**; *H. geographicus ***(d)**; *H. multifasciatus ***(e)**; *H. raniceps ***(f)**; and *H. wavrini ***(g)**. The chromosome pairs bearing the nucleolus organizer regions are identified in the corresponding boxes.

**Figure 3:**
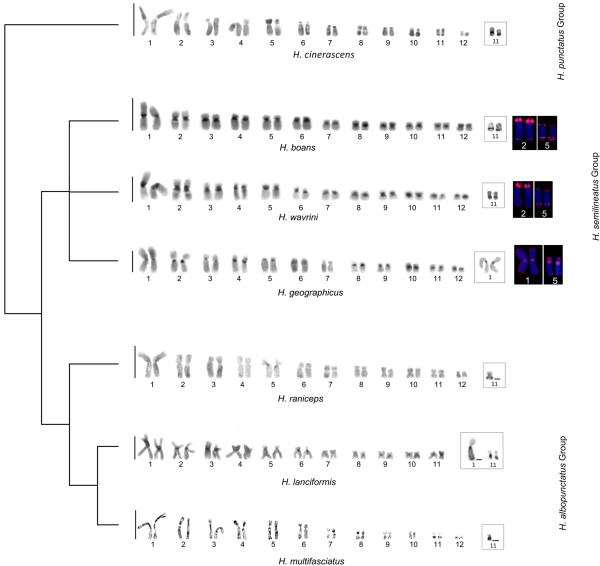
**Partial phylogenetic diagram proposed by Faivovich *et al. ***[13]**for *Hypsiboas*, including the cytogenetic data.** Emphasis is on the relationships between the *H. punctatus*, *H. semilineatus,* and *H. albopunctatus* groups*.* The lack of definition of the *H. semilineatus* group branches is a result of *H. wavrini* not having been included in the original phylogeny (not all representative species of the *H. semilineatus* group were included).

Different distribution patterns of constitutive heterochromatin were observed in the *Hypsiboas* species analyzed. Heterochromatin was distributed preferentially in the centromeric regions of most of the chromosomes of *H. lanciformis*, with some blocks invading the pericentromeric region, sometimes including the entire short arm, while other chromosomes showed no evident heterochromatin (Figure 2a). Large conspicuous blocks of constitutive heterochromatin in the centromeric and pericentromeric regions of all the chromosomes were present in *H. boans* (Figure 2b), *H. geographicus* (Figure 2d), and *H. wavrini* (Figure 2g), with the exception of the pairs 6 and 7 of the homologs of pairs 6 and 7 of *H. geographicus*, which did not show any heterochromatic blocks. The heterochromatic portions of *H. cinerascens* were poorly distinguishable (Figure 2c), although some pairs were clearly defined in the centromeric region as seen in pairs 1, 2, 3, 5, 6, and 8 (Figure 2c). The C-banding in *H. multifasciatus* showed interstitial distributions along the short and long arms of most of the chromosomes, as well as on the long arms of pairs 11 and 12 (Figure 2e). Constitutive heterochromatin was absent from most of the chromosomes of *H. raniceps*, although conspicuous heterochromatic blocks occurred in the pericentromeric regions of pair 5 (Figure 2f). In this study, the heterochromatin data of three species in the *H. semilineatus* group distinguished them from four species in the other groups (Figure 3).

*Hypsiboas lanciformis* had multiple NORs, with one centromeric mark in only one of the chromosomes of pair 1 and in the subterminal region of the long arm of pair 11 (box in Figure 2a). For the other species we investigated, a single chromosome pair was stained by the AgNO_3_. The silver‒binding NORs were primarily located on chromosomal pair 11 in *H. boans*, *H. cinerascens*, *H. multifasciatus*, *H. raniceps*, and *H. wavrini* (boxes in Figures 2b, c, e–g) and on the centromeric region of pair 1 in *H. geographicus* (box in Figure 2d). Variations in the number of active sites were observed among and within individuals of all species.

### Telomeric sequence mapping

**Figure 4:**
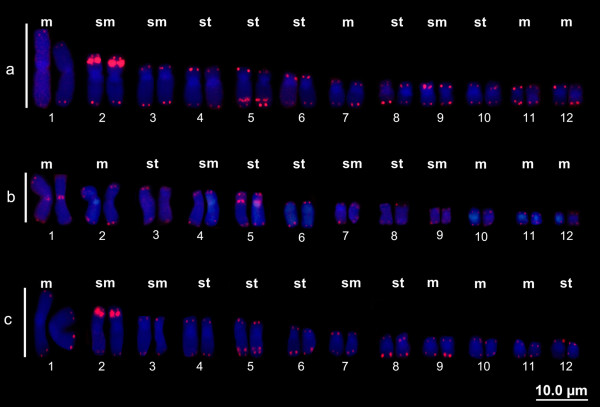
**Telomeric hybridization**. *Hypsiboas boans ***(a)**, *H. geographicus ***(b)**, and *H. wavrini ***(c)**, which are in the *H. semilineatus* group, showed signs of hybridization based on the telomeric probe (red). The chromosomes were counterstained with DAPI.

Combining telomeric probes with fluorescence in situ hybridization (FISH) detected sites in the terminal regions of all of the chromosomes of all species. Interstitial telomeric sites (ITSs) were detected in three of the species belonging to the *H. semilineatus* group: *H. boans*, *H. geographicus*, and *H. wavrini.* In *H. boans* and *H. wavrini* (Figures 4a and c, respectively), the ITSs were seen on the short arms of both homologs of pair 2 and on the long arms of both homologs of pair 5. The centromeric ITSs in *H. geographicus* were seen on both homologs of pairs 1 and 5; the ITSs on pair 1 correspond with the NOR sites in this species (Figure 4b). However, ITSs were not found in *H. cinerascens*, *H. lanciformis*, *H. multifasciatus* and *H. raniceps*.

## Discussion

The diploid chromosome number in the species of the family Hylidae varies between 18 and 30. Species of *Dendropsophus* have 2n = 30 chromosomes [3], those in *Phyllomedusa* have 2n = 26 chromosomes [35], *Hyla* have 2n = 24 chromosomes [36,37], *Aplastodiscus* has diploid numbers ranging from 18 to 24 [6], and most of the *Hypsiboas* species have 2n = 24 chromosomes [10,18,28].

The diploid number of 22 can be seen in *H. albopunctatus*[4,9] and *H. lanciformis* [present work], both of which are in the *H. albopunctatus* group. Based on the chromosome data of *H. albopunctatus*, it is possible that an end-to-end fusion occurred in the *Hypsiboas* ancestral (2n = 24) involving small chromosomes, probably chromosome pairs 11 (NOR region) and 12, because they are similar in length [4]. The same may also have occurred in *H. lanciformis*, explaining the evolution of the karyotype of this species with its reduced diploid number.

ITSs are repetitive sequences, which can derive from chromosomal rearrangements (centric fusion, *in tandem* fusion, or inversion) during vertebrate karyotype evolution [38,39] representing the remaining sequences in newly formed chromosomes. Alternatively, ITSs can also result from the amplification of telomeric sequences, be the result of unequal crossing-over and transposition, be sequences introduced by a telomerase error, or be the result of integration between transposons and telomeric sequences [37,38]. ITSs have been found in hylid frogs and in some species of *Hyla* in North America such as *H. chrysoscelis* and *H. versicolor*; these have been attributed to unequal crossing-over during meiosis, submicroscopic deletions, and differential amplifications [37,40]. Telomeric sequences commonly occur outside of the terminal regions in the Hylidae family [37,38] and are present in *H. boans*, *H. geographicus*, and *H. wavrini* belong to *H. semilineatus* group. These sequences may be the result of chromosomal rearrangements and represent the remains of sequences in newly formed chromosomes, or they may be due to integration between transposons and telomeric sequences as has been observed in other species [37,38]. There is no consensus on the presence or absence of ITSs and their relation to chromosomal rearrangements, because many factors may be involved [41]. However, no ITSs were found in the other four *Hypsiboas* species analyzed in this study, suggesting the answer may be selection by an unknown agent that may not alter their fitness [37].

The absence of the ITSs in most *Hypsiboas* species does not necessarily indicate that the hypothesis that *Hypsiboas* species were derived from a common ancestor with 26 chromosomes is incorrect. Chromosomes derived from fusion events may have small telomeric sites that cannot be easily detected by FISH, or the telomeres could be lost before the fusion or eroded by molecular processes [42]. As there has been no alteration in the basal diploid number of *Hypsiboas*, the ITSs observed in the chromosomes of *H. boans*, *H. geographicus*, and *H. wavrini* probably reflect non-Robertsonian rearrangements, given that that these three species are not found in the basal group.

*Hypsiboas cinerascens* (previously *Hyla granosa* within the *Hyla granosa* group) belongs to the *Hypsiboas punctatus* group (monophyletic group fusion between the *Hyla punctata* and *Hyla granosa* groups) [13] and displays the basal cytogenetic characteristics of the *Hypsiboas*, including a diploid number of 24, poorly visible constitutive heterochromatin, and an active NOR on a single chromosome pair [14, present work]. A detailed comparison between the karyotypic patterns of the *Hypsiboas punctatus* group was not possible, because the data was restricted to the *Hypsiboas punctatus* diploid number of 24 [14,21,29,30].

The species *H. boans*, *H. geographicus*, and *H. wavrini* (*H. semilineatus* group) show processes of heterochromatin accumulation or heterochromatization [43-45] during their evolution, a characteristic that distinguishes these species from the others in the group. Additionally, *H. boans* and *H. wavrini* are phylogenetically related, with similar patterns of constitutive heterochromatin distribution, and the number and localization of the NORs and ITSs. The proximity between *H. wavrini* and *H. boans* can also be seen in their morphological and reproductive similarities, which makes it difficult to differentiate between these species in the field [46,47]. Both species have been found in sympatry in Colombia, and though they occupy identical niches, they differ in their vocalization and reproductive periods [48]. However, the karyotype formulas differ between *H. boans* and *H. wavrini*, allowing them to be differentiated [present work].

Almost 90% of speciation events are accompanied by chromosome changes [42]. NORs are considered excellent markers in karyotype evolution studies in amphibians [49,50], despite the occurrence of rare variations within the species [51]. Most anuran genera have heteromorphic NORs, and the differences in their size may be due to *in tandem* duplication or triplication, which can affect one or both DNAr clusters [49]. The duplicated NORs found in *H. albomarginatus* may have resulted from differential gene activity or be a duplication by mobile elements [47]. In one study, three of four species (*H. albomarginatus*, *H. semilineatus*, and *H. pardalis*) had heteromorphic NORs [17].

*Hypsiboas geographicus* (*H. semilineatus* group) had NORs present on the centromeric region of pair 1, while in the other species of this group, the NORs were present in another chromosomes pair, such as pair 11 in *H. semilineatus*[17]. In *H. geographicus*, the NOR is in same region as the ITS, and it is possible that both structures are associated with different satellite/repetitive DNA classes, because they are in the centromeric region [52]. Despite some authors being unable to find an association between the NORs (specifically 18S rDNA) and ITSs [37], this would explain the amplification of those telomeric sequences in the interstitial region of the chromosomes. However, the silver nitrate impregnation technique only identifies active sites [53], meaning the possibility of multiple ribosomal sites in these groups or variable chromosomal localization among the species cannot be eliminated. Multiple NOR active sites were observed in *H. lanciformis* [present work] and *H. raniceps*[4] (both in the *H. albopunctatus* group) [13], as well as in *H. prasinus* (*H. pulchellus* group) [9,23,27]. The hybridization of 45S ribosomal DNA probes only in *H. albopunctatus* and *H. pardalis* indicated the presence of one labeled chromosome pair [14,17]. Since silver associates with nucleolar proteins involved in the transcriptional activity of ribosomal genes from the 45S rDNA cistrons [51,52] and can also impregnate heterochromatic regions rich in acidic residues [54], the multiple NORs present in *H. lanciformis* and *H. raniceps* may be indicating a heterochromatic region. However, the position of the NORs varies among the species, and it is possible that the ribosomal genes are changing during the karyotypic evolutionary process [17,48,49]. Despite some authors [17] finding that each monophyletic clade in the Hylidae phylogenetic tree [13] had the ribosomal cistron located in a specific chromosome pair (based on the NOR data), there may be no typical pattern for each group, with the presence of both simple and multiple NORs among the species of those taxa (Table 1).

In addition to the differences in the number and localization of the NORs, two different diploid numbers and different karyotypic formulas were seen in the *H. albopunctatus* group. As such, in spite of the fact that *H. albopunctatus* has 2n = 22 chromosomes, other species of the group have a diploid number of 24 [4,9,10,12,15], and this reduction to 22 is not a true characteristic of the group. In addition, despite the decrease to 2n = 22 chromosomes in *H. lanciformis*, no ITSs were encountered in that species, possibly due to genetic erosion of those sequences. Given that *H. lanciformis* is typically found in forest fragments and along forest edges [55-57] where it would be more susceptible to anthropogenic interactions such as water contamination, which can cause several diseases [58], the lack of ITSs could also be due to selection by an unknown agent that does not prejudice the development of the species [37,41]. These same forces may also be acting on *H. albopunctatus*, which frequently occurs in disturbed areas [57]; in addition to having 22 chromosome pairs, many individuals of this species have supernumerary chromosomes [4].

Both species, *Hypsiboas lanciformis* [present work] and *H. albopunctatus*[4,9], had a reduced chromosome number (2n = 22) relative to their co-generic species (2n = 24) in a phylogenetic tree of the family Hylidae [13]. However, looking at the diploid number data plotted for this tree, it is apparent that chromosome number can either occur independently in these species, is related to their natural history, or is a species characteristic [43]. Thus, they have the same common ancestor, but are grouped in different clades [13].

Different patterns of distribution of the heterochromatin were found in the *H. albopunctatus* group. *H. lanciformis* had heterochromatic blocks in the centromeric region of most of its chromosomes [present work], as did *H. albopunctatus*[4,12]. *H. raniceps* and *H. multifasciatus* showed weak heterochromatic blocks distributed in only a few pairs of chromosomes. In addition, there were clear differences in the distribution patterns of heterochromatin among the populations of *H. raniceps* such as between the individuals from the northern and central regions of Brazil [4,10,11,15]. The variation in the quantity and distribution of the constitutive heterochromatin is an important characteristic that can be used to differentiate between populations based on an epigenetic mechanism [59]. Additionally, heterochromatin is normally rich in repetitive sequences that may have important roles in speciation and/or adaptation, as they are less subject to selective pressure—which favors the accumulation of differences during evolutive processes [44,60,61].

Differences in genome size are primarily due to events of heterochromatin addition or deletion involving DNA satellite families [62]. The DNA content was 6.61 pg/N for *H. lanciformis*, while those of *H. cinerascens* (synonym of *Hyla granosa*) and *Hypsiboas geographicus* were 4.53 pg/N and 3.28 pg/N [45], respectively. Heterochromatin was present in the centromeric region in most of the chromosomes of both *H. lanciformis* and *H. cinerascens*, but like the DNA content, the quantity of heterochromatin was different relative to others such as *H. lanciformis*, which had more heterochromatic blocks and a higher DNA content. However, when the C-banding patterns of *H. lanciformis* and *H. geographicus* were compared, their heterochromatin was similar, despite the difference in their DNA content. Recent work has demonstrated epigenetic influences on the pattern of heterochromatin distribution in chromosomes [59,63] that could explain the absence of a correlation between the high DNA content and more heterochromatic blocks found in *H. geographicus* and the number and localization of NORs and ITSs.

## Conclusion

The data presented in this study reinforces the complexity previously observed within the genus *Hypsiboas* and in the different groups that compose this taxon. More studies focusing on this group and covering larger sampling areas, especially in the Brazilian Amazon, are needed to gain a better understanding of this fascinating, but complex group.

## Methods

### Species and collection localities

The collections were undertaken between June 2011 and June 2012, during both the rainy and dry seasons under the authorization of the Instituto Chico Mendes de Conservação da Biodiversidade (11323–0). This work was authorized by the Ethics Committee of Animal Experimentation (CEEA) of the Amazonas Federal University (no. 075/2012). Voucher specimens were deposited in the Paulo Bührnheim Zoological Collection of the Amazonas Federal University (CZPB/UFAM) and the Collection of Amphibians and Reptiles of the National Institute of Amazonian Research (INPA-H).

Twenty-two specimens were analyzed: 1 male *H. boans* (INPA-H 314433), collected in São Sebastião do Uatumã (AM) (0°50' to 1°55'S; 58°50' to 60°10'W); 4 male *H. cinerascens* (CZPB/UFAM 153/315, CZPB/UFAM 154/316–318), collected in Manaus (AM) (03°04'34"S; 59°57'30"W); 1 female and 3 male *H. geographicus* (INPA-H 31445, INPA-H 31447–31448, INPA-H 31450), collected in Santa Isabel do Rio Negro (AM) (0°24'24"N; 65°1'1"W); 3 male *H. lanciformis* (CZPB/UFAM 155/319,CZPB/UFAM 159/331,CZPB/UFAM 159/333), collected in Manaus (AM) (03°04'34"S; 59°57'30"W); 1 female *H. multifasciatus* (CZPB/UFAM 156/320), collected in Iranduba (AM) (03°09'47"S; 59°54'29"W); 2 female and 4 male *H. raniceps* (CZPB/UFAM 158/324–329), collected in Iranduba (AM) (03°09'47"S; 59°54'29"W); 1 female *H. wavrini*, collected in São Sebastião do Uatumã (AM) (0°50' to 1°55'S; 58°50' to 60°10'W), and 2 male *H. wavrini* (INPA-H 31441–31442; INPA-H 31444), collected in Santa Isabel do Rio Negro (AM) (0°24'24"N; 65°1'1"W).

### Chromosomal analyses

Mitotic chromosomes were obtained from the bone marrow and liver of the hylids after in vitro (1%) and in vivo (0.1%) colchicine exposure [64,65]. Cell division was induced in some specimens by injecting them with biological yeast (0.1 mL per 10 g of animal bodyweight) and maintaining them alive for 48–72 h [35,66].

### Classical and molecular cytogenetic analysis

The cell suspensions were analyzed after routine staining with conventional stain (10%), C-banding [54], Ag-NOR staining [53], and FISH [67] with a telomeric probe. The FISH telomeric probe was digoxigenin-labelled by a nick translation reaction using a Roche^TM^ kit and amplified by polymerase chain reactions as previously described [68]. Chromosomes were organized by decreasing size, and the morphology was determined based on the centromere position [69]. Fundamental numbers were determined by conventional staining at metaphase and by exposure to barium hydroxide.

## Abbreviations

FISH: Fluorescence in situ hybridization; ITS: Interstitial telomeric site; NOR: Nucleolar organizer region.

## Competing interests

The authors declare that they have no competing interests.

## Authors’ contributions

TLM, ACC, CHS, and DOCT collected the samples and analyzed the results. TLM and ACC collaborated on all genetic procedures, undertook the bibliographic review, and coordinated the writing of the paper. CHS participated in developing the molecular cytogenetics techniques with TLM and analyzing the results. MCG and MM coordinated the study, participated in its design, analyzed the results, and revised the manuscript. All authors read and approved the final manuscript.
